# 7-Phenyl­sulfonyl-7*H*-benzofurano[2,3-*b*]carbazole

**DOI:** 10.1107/S1600536811039705

**Published:** 2011-10-05

**Authors:** R. Panchatcharam, V. Dhayalan, A. K. Mohanakrishnan, G. Chakkaravarthi, V. Manivannan

**Affiliations:** a Centre for Research and Development, PRIST University, Vallam, Thanjavur 613 403, Tamil Nadu, India; b Department of Organic Chemistry, University of Madras, Guindy Campus, Chennai 600 025, India; c Department of Physics, CPCL Polytechnic College, Chennai 600 068, India

## Abstract

In the title compound, C_24_H_15_NO_3_S, the dihedral angle between the phenyl ring and the carbozole system is 74.91 (6)°. The S atom exhibits a distorted tetra­hedral geometry [N—S—C = 104.85 (8)°; O—S—O = 119.59 (9)°]. The crystal structure is established by weak inter­molecular π–π inter­actions [centroid–centroid distances = 3.583 (2)–3.782 (2) Å].

## Related literature

For the biological activity of carbazole derivatives, see: Ramsewak *et al.* (1999 ▶); Tachibana *et al.* (2001 ▶). For the structures of closely related compounds, see: Chakkaravarthi *et al.* (2008*a* ▶,*b* ▶).
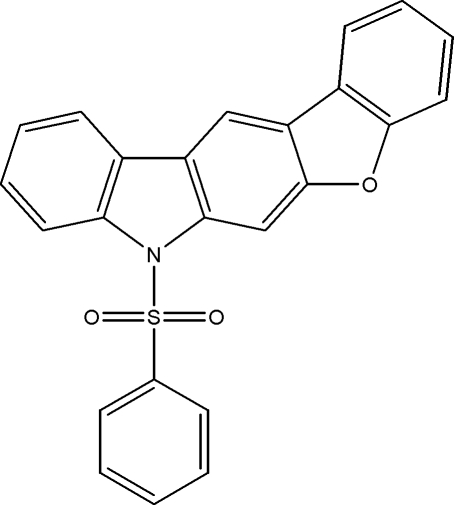


## Experimental

### 

#### Crystal data

C_24_H_15_NO_3_S
*M* *_r_* = 397.43
Monoclinic, 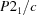

*a* = 9.031 (5) Å
*b* = 10.752 (6) Å
*c* = 19.217 (5) Å
β = 100.738 (5)°
*V* = 1833.3 (15) Å^3^
*Z* = 4
Mo *K*α radiation
μ = 0.20 mm^−1^
*T* = 295 K
0.26 × 0.22 × 0.20 mm

#### Data collection

Bruker Kappa APEXII diffractometer
Absorption correction: multi-scan (*SADABS*; Sheldrick, 1996 ▶) *T*_min_ = 0.949, *T*_max_ = 0.960
16640 measured reflections
4462 independent reflections
2763 reflections with *I* > 2σ(*I*)
*R*_int_ = 0.027

#### Refinement

*R*[*F* ^2^ > 2σ(*F* ^2^)] = 0.041
*wR*(*F* ^2^) = 0.112
*S* = 1.04
4462 reflections
262 parameters
1 restraint
H-atom parameters constrained
Δρ_max_ = 0.23 e Å^−3^
Δρ_min_ = −0.30 e Å^−3^

### 

Data collection: *APEX2* (Bruker, 2004 ▶); cell refinement: *SAINT* (Bruker, 2004 ▶); data reduction: *SAINT*; program(s) used to solve structure: *SHELXS97* (Sheldrick, 2008 ▶); program(s) used to refine structure: *SHELXL97* (Sheldrick, 2008 ▶); molecular graphics: *PLATON* (Spek, 2009 ▶); software used to prepare material for publication: *SHELXL97*.

## Supplementary Material

Crystal structure: contains datablock(s) global, I. DOI: 10.1107/S1600536811039705/im2318sup1.cif

Structure factors: contains datablock(s) I. DOI: 10.1107/S1600536811039705/im2318Isup2.hkl

Supplementary material file. DOI: 10.1107/S1600536811039705/im2318Isup3.cml

Additional supplementary materials:  crystallographic information; 3D view; checkCIF report

## Figures and Tables

**Fig. 1.**
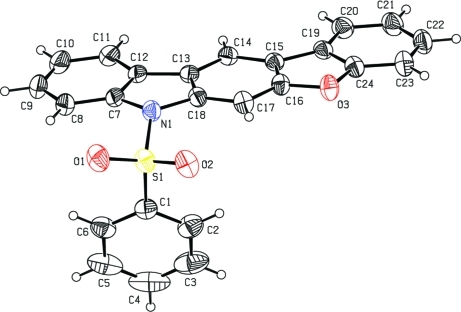
Molecular structure of (I), with atom labels and 30% probability displacement ellipsoids for non-H atoms.

## supplementary crystallographic information

### Comment

In continuation of our studies of carbazole derivatives, which are found to possess various biological activities such as antioxidative (Tachibana *et al.*, 2001), anti-inflammatory and antimutagenic (Ramsewak *et al.*, 1999), we report the crystal structure of the title compound (I) (Fig. 1). The geometric parameters of (I) are agree well with similar reported structures [Chakkaravarthi *et al.* 2008*a*, 2008*b*].

The dihedral angle beween the phenyl ring (C1-C6) and the carbozole ring (N1/C7-C18) is 74.91 (6)°. The benzofuran moiety (C15/C19-C24/O3/C16) is almost co-planar [dihedral angle 2.48 (3)°] with the carbazole ring system. In the molecule, the S atom exhibits a distorted tetrahedral [N1-S1-C1 = 104.85 (8)°; O1-S1-O2 = 119.59 (9)°] geometry .

The crystal structure is established by weak intermolecular π–π interactions [Cg1···Cg6 (-x,1-y,1-z) = 3.583 (2) Å; Cg2···Cg6 (1-x,1-y,1-z) = 3.782(2 )Å; Cg4···Cg6 (1-x,1-y,1-z) = 3.730 (2) Å; Cg6···Cg6 (-x,1-y,1-z) = 3.659(2 )Å; Cg1, Cg2, Cg4 and Cg6 are the centroids of the rings (O3/C16/C15/C19/C24), (N1/C7/C12/C13/C18), (C7-C12) and (C19-C24), respectively].

### Experimental

To a solution of diethyl-2-((2-(bromomethyl)-1-(phenylsulfonyl)-1H-indol-3-yl) methylene)malonate (0.2 g, 0.38 mmol) in anhydrous 1,2-dichloroethane (15 mL), anhydrous ZnBr_2_ (0.02 g, 0.08 mmol) and benzo[b]furan (0.04 mL, 0.38 mmol) were added. The mixture was then stirred at room temperature for 2 h under N_2_ atmosphere. After the solvent was removed,and the residue was quenched with ice-water (50 mL) containing 1 mL of conc.HCl, extracted with chloroform (2 x 10 mL) and dried (Na_2_SO_4_). Removal of solvent followed by flash column chromatography (n-hexane) led to the isolation of colourless crystals suitable for X-ray diffraction quality after the solvent was evaporated at room temperature (yield: 0.11 g, 73%).

### Refinement

All H atoms were positioned geometrically with C—H = 0.93Å, and allowed to ride on their parent atoms, with U_iso_(H) = 1.2 U_eq_(C). The anisotropic displacement in the direction of bond C19 and C24 were restrained to be equal within an effective standard deviation of 0.001 using the DELU command in the final cycles of refinement (Sheldrick, 2008).

### Crystal data

**Table:**

C_24_H_15_NO_3_S	*F*(000) = 824
*M**_r_* = 397.43	*D*_x_ = 1.440 Mg m^−^^3^
Monoclinic, *P*2_1_/*c*	Mo *K*α radiation, λ = 0.71073 Å
Hall symbol: -P 2ybc	Cell parameters from 4232 reflections
*a* = 9.031 (5) Å	θ = 2.2–28.3°
*b* = 10.752 (6) Å	µ = 0.20 mm^−^^1^
*c* = 19.217 (5) Å	*T* = 295 K
β = 100.738 (5)°	Block, colourless
*V* = 1833.3 (15) Å^3^	0.26 × 0.22 × 0.20 mm
*Z* = 4	

### Data collection

**Table:**

Bruker Kappa APEXII diffractometer	4462 independent reflections
Radiation source: fine-focus sealed tube	2763 reflections with *I* > 2σ(*I*)
graphite	*R*_int_ = 0.027
ω and φ scans	θ_max_ = 28.3°, θ_min_ = 2.2°
Absorption correction: multi-scan (*SADABS*; Sheldrick, 1996)	*h* = −11→12
*T*_min_ = 0.949, *T*_max_ = 0.960	*k* = −12→14
16640 measured reflections	*l* = −25→24

### Refinement

**Table:**

Refinement on *F*^2^	Primary atom site location: structure-invariant direct methods
Least-squares matrix: full	Secondary atom site location: difference Fourier map
*R*[*F*^2^ > 2σ(*F*^2^)] = 0.041	Hydrogen site location: inferred from neighbouring sites
*wR*(*F*^2^) = 0.112	H-atom parameters constrained
*S* = 1.04	*w* = 1/[σ^2^(*F*_o_^2^) + (0.0453*P*)^2^ + 0.360*P*] where *P* = (*F*_o_^2^ + 2*F*_c_^2^)/3
4462 reflections	(Δ/σ)_max_ < 0.001
262 parameters	Δρ_max_ = 0.23 e Å^−^^3^
1 restraint	Δρ_min_ = −0.30 e Å^−^^3^

### Fractional atomic coordinates and isotropic or equivalent isotropic displacement parameters (Å^2^)

**Table:**

	*x*	*y*	*z*	*U*_iso_*/*U*_eq_	
C1	0.2652 (2)	0.06505 (18)	0.27600 (9)	0.0556 (5)	
C2	0.1266 (2)	0.1058 (2)	0.28782 (11)	0.0751 (6)	
H2	0.1027	0.1900	0.2854	0.090*	
C3	0.0258 (3)	0.0206 (4)	0.30312 (14)	0.1056 (10)	
H3	−0.0677	0.0471	0.3109	0.127*	
C4	0.0599 (4)	−0.1031 (4)	0.30715 (13)	0.1126 (12)	
H4	−0.0105	−0.1603	0.3173	0.135*	
C5	0.1988 (4)	−0.1433 (3)	0.29616 (13)	0.0992 (9)	
H5	0.2224	−0.2276	0.2994	0.119*	
C6	0.3021 (3)	−0.0594 (2)	0.28043 (11)	0.0713 (6)	
H6	0.3958	−0.0861	0.2729	0.086*	
C7	0.56766 (19)	0.14212 (16)	0.38858 (10)	0.0522 (5)	
C8	0.6548 (2)	0.04173 (18)	0.37621 (13)	0.0661 (6)	
H8	0.6619	0.0176	0.3305	0.079*	
C9	0.7310 (2)	−0.0212 (2)	0.43485 (15)	0.0789 (7)	
H9	0.7893	−0.0900	0.4283	0.095*	
C10	0.7227 (2)	0.0153 (2)	0.50279 (14)	0.0761 (6)	
H10	0.7770	−0.0279	0.5412	0.091*	
C11	0.6352 (2)	0.11476 (18)	0.51438 (12)	0.0641 (5)	
H11	0.6297	0.1391	0.5603	0.077*	
C12	0.55535 (19)	0.17832 (16)	0.45685 (10)	0.0498 (4)	
C13	0.45251 (18)	0.28238 (15)	0.45167 (9)	0.0464 (4)	
C14	0.40052 (19)	0.35208 (15)	0.50305 (10)	0.0487 (4)	
H14	0.4316	0.3350	0.5510	0.058*	
C15	0.30071 (18)	0.44786 (15)	0.48026 (9)	0.0450 (4)	
C16	0.2592 (2)	0.47184 (15)	0.40826 (9)	0.0489 (4)	
C17	0.3092 (2)	0.40679 (16)	0.35562 (10)	0.0534 (5)	
H17	0.2806	0.4264	0.3079	0.064*	
C18	0.40576 (19)	0.30960 (15)	0.37963 (9)	0.0473 (4)	
C19	0.21968 (19)	0.53778 (15)	0.51514 (10)	0.0485 (4)	
C20	0.2091 (2)	0.56435 (19)	0.58450 (10)	0.0620 (5)	
H20	0.2654	0.5200	0.6218	0.074*	
C21	0.1136 (2)	0.6577 (2)	0.59692 (12)	0.0690 (6)	
H21	0.1056	0.6764	0.6433	0.083*	
C22	0.0298 (2)	0.7241 (2)	0.54246 (12)	0.0693 (6)	
H22	−0.0343	0.7864	0.5527	0.083*	
C23	0.0385 (2)	0.70030 (18)	0.47297 (12)	0.0656 (5)	
H23	−0.0179	0.7450	0.4358	0.079*	
C24	0.1350 (2)	0.60726 (16)	0.46146 (10)	0.0530 (4)	
N1	0.47915 (16)	0.22546 (13)	0.33891 (8)	0.0525 (4)	
O1	0.50962 (17)	0.11440 (14)	0.22963 (8)	0.0782 (4)	
O2	0.31857 (18)	0.27874 (12)	0.22364 (7)	0.0715 (4)	
O3	0.15849 (15)	0.57003 (11)	0.39524 (7)	0.0602 (4)	
S1	0.39665 (6)	0.17516 (5)	0.25941 (2)	0.05766 (16)	

### Atomic displacement parameters (Å^2^)

**Table:**

	*U*^11^	*U*^22^	*U*^33^	*U*^12^	*U*^13^	*U*^23^
C1	0.0605 (11)	0.0645 (12)	0.0413 (10)	−0.0043 (10)	0.0086 (9)	0.0010 (9)
C2	0.0644 (13)	0.0969 (17)	0.0653 (14)	−0.0057 (12)	0.0157 (11)	−0.0002 (12)
C3	0.0810 (18)	0.158 (3)	0.0819 (19)	−0.047 (2)	0.0259 (15)	−0.0159 (19)
C4	0.129 (3)	0.150 (3)	0.0561 (15)	−0.087 (3)	0.0089 (16)	−0.0030 (17)
C5	0.139 (3)	0.0823 (17)	0.0648 (16)	−0.0455 (19)	−0.0103 (17)	0.0090 (13)
C6	0.0848 (15)	0.0645 (13)	0.0602 (13)	−0.0085 (12)	0.0019 (11)	−0.0012 (10)
C7	0.0395 (9)	0.0463 (9)	0.0719 (13)	−0.0024 (8)	0.0132 (9)	−0.0024 (9)
C8	0.0517 (11)	0.0588 (12)	0.0893 (16)	0.0051 (10)	0.0176 (11)	−0.0117 (11)
C9	0.0531 (12)	0.0608 (13)	0.120 (2)	0.0159 (10)	0.0103 (14)	−0.0057 (14)
C10	0.0565 (13)	0.0655 (13)	0.0987 (19)	0.0121 (11)	−0.0048 (12)	0.0068 (13)
C11	0.0524 (11)	0.0604 (12)	0.0752 (14)	0.0034 (10)	0.0003 (10)	0.0000 (11)
C12	0.0392 (9)	0.0440 (9)	0.0650 (12)	−0.0040 (8)	0.0069 (9)	−0.0012 (9)
C13	0.0418 (9)	0.0418 (9)	0.0558 (11)	−0.0055 (7)	0.0098 (8)	0.0002 (8)
C14	0.0491 (10)	0.0473 (10)	0.0490 (10)	−0.0033 (8)	0.0071 (8)	−0.0003 (8)
C15	0.0452 (9)	0.0417 (9)	0.0498 (10)	−0.0049 (8)	0.0135 (8)	−0.0017 (8)
C16	0.0524 (10)	0.0423 (9)	0.0552 (11)	0.0031 (8)	0.0184 (9)	0.0071 (8)
C17	0.0645 (11)	0.0503 (10)	0.0486 (11)	0.0042 (9)	0.0188 (9)	0.0075 (8)
C18	0.0484 (10)	0.0423 (9)	0.0550 (11)	−0.0011 (8)	0.0191 (8)	−0.0002 (8)
C19	0.0474 (10)	0.0426 (9)	0.0576 (10)	−0.0065 (7)	0.0153 (8)	−0.0034 (7)
C20	0.0657 (12)	0.0640 (12)	0.0568 (12)	−0.0010 (10)	0.0128 (10)	−0.0071 (10)
C21	0.0743 (14)	0.0698 (13)	0.0676 (14)	0.0015 (11)	0.0248 (12)	−0.0170 (11)
C22	0.0698 (13)	0.0582 (12)	0.0861 (16)	0.0087 (10)	0.0305 (12)	−0.0119 (11)
C23	0.0705 (13)	0.0547 (11)	0.0756 (14)	0.0148 (10)	0.0245 (11)	0.0038 (10)
C24	0.0590 (11)	0.0453 (10)	0.0589 (11)	−0.0005 (8)	0.0222 (8)	0.0000 (8)
N1	0.0514 (8)	0.0495 (8)	0.0597 (9)	0.0029 (7)	0.0188 (8)	−0.0034 (7)
O1	0.0858 (10)	0.0817 (10)	0.0794 (10)	0.0048 (8)	0.0476 (8)	−0.0124 (8)
O2	0.1036 (11)	0.0625 (8)	0.0527 (8)	0.0097 (8)	0.0257 (8)	0.0121 (7)
O3	0.0742 (9)	0.0538 (7)	0.0562 (8)	0.0178 (7)	0.0216 (7)	0.0103 (6)
S1	0.0692 (3)	0.0567 (3)	0.0535 (3)	0.0019 (2)	0.0282 (2)	0.0007 (2)

### Geometric parameters (Å, °)

**Table:**

C1—C6	1.378 (3)	C13—C18	1.401 (2)
C1—C2	1.385 (3)	C14—C15	1.385 (2)
C1—S1	1.747 (2)	C14—H14	0.9300
C2—C3	1.362 (4)	C15—C16	1.389 (2)
C2—H2	0.9300	C15—C19	1.450 (2)
C3—C4	1.364 (5)	C16—C17	1.373 (2)
C3—H3	0.9300	C16—O3	1.385 (2)
C4—C5	1.380 (4)	C17—C18	1.384 (2)
C4—H4	0.9300	C17—H17	0.9300
C5—C6	1.372 (3)	C18—N1	1.436 (2)
C5—H5	0.9300	C19—C24	1.383 (3)
C6—H6	0.9300	C19—C20	1.384 (3)
C7—C8	1.382 (3)	C20—C21	1.373 (3)
C7—C12	1.392 (3)	C20—H20	0.9300
C7—N1	1.438 (2)	C21—C22	1.372 (3)
C8—C9	1.383 (3)	C21—H21	0.9300
C8—H8	0.9300	C22—C23	1.376 (3)
C9—C10	1.379 (3)	C22—H22	0.9300
C9—H9	0.9300	C23—C24	1.371 (3)
C10—C11	1.372 (3)	C23—H23	0.9300
C10—H10	0.9300	C24—O3	1.388 (2)
C11—C12	1.383 (3)	N1—S1	1.6606 (16)
C11—H11	0.9300	O1—S1	1.4187 (14)
C12—C13	1.446 (2)	O2—S1	1.4246 (15)
C13—C14	1.389 (2)		
			
C6—C1—C2	120.9 (2)	C13—C14—H14	121.2
C6—C1—S1	120.30 (17)	C14—C15—C16	119.48 (16)
C2—C1—S1	118.76 (17)	C14—C15—C19	134.79 (17)
C3—C2—C1	118.9 (3)	C16—C15—C19	105.73 (15)
C3—C2—H2	120.6	C17—C16—O3	123.35 (16)
C1—C2—H2	120.6	C17—C16—C15	125.07 (16)
C2—C3—C4	121.1 (3)	O3—C16—C15	111.57 (15)
C2—C3—H3	119.5	C16—C17—C18	114.34 (17)
C4—C3—H3	119.5	C16—C17—H17	122.8
C3—C4—C5	119.9 (3)	C18—C17—H17	122.8
C3—C4—H4	120.1	C17—C18—C13	122.85 (16)
C5—C4—H4	120.1	C17—C18—N1	128.31 (16)
C6—C5—C4	120.2 (3)	C13—C18—N1	108.79 (15)
C6—C5—H5	119.9	C24—C19—C20	118.62 (17)
C4—C5—H5	119.9	C24—C19—C15	105.78 (16)
C5—C6—C1	119.1 (2)	C20—C19—C15	135.59 (18)
C5—C6—H6	120.5	C21—C20—C19	118.47 (19)
C1—C6—H6	120.5	C21—C20—H20	120.8
C8—C7—C12	121.89 (19)	C19—C20—H20	120.8
C8—C7—N1	129.50 (18)	C22—C21—C20	121.5 (2)
C12—C7—N1	108.60 (15)	C22—C21—H21	119.2
C7—C8—C9	117.0 (2)	C20—C21—H21	119.2
C7—C8—H8	121.5	C21—C22—C23	121.32 (19)
C9—C8—H8	121.5	C21—C22—H22	119.3
C10—C9—C8	121.7 (2)	C23—C22—H22	119.3
C10—C9—H9	119.1	C24—C23—C22	116.4 (2)
C8—C9—H9	119.1	C24—C23—H23	121.8
C11—C10—C9	120.7 (2)	C22—C23—H23	121.8
C11—C10—H10	119.7	C23—C24—C19	123.60 (18)
C9—C10—H10	119.7	C23—C24—O3	124.67 (17)
C10—C11—C12	119.0 (2)	C19—C24—O3	111.72 (15)
C10—C11—H11	120.5	C18—N1—C7	106.65 (14)
C12—C11—H11	120.5	C18—N1—S1	122.20 (12)
C11—C12—C7	119.59 (17)	C7—N1—S1	120.42 (12)
C11—C12—C13	132.08 (18)	C16—O3—C24	105.18 (14)
C7—C12—C13	108.34 (16)	O1—S1—O2	119.59 (9)
C14—C13—C18	120.67 (16)	O1—S1—N1	106.74 (9)
C14—C13—C12	131.80 (17)	O2—S1—N1	106.63 (8)
C18—C13—C12	107.53 (15)	O1—S1—C1	109.01 (10)
C15—C14—C13	117.56 (17)	O2—S1—C1	109.01 (10)
C15—C14—H14	121.2	N1—S1—C1	104.85 (8)
			
C6—C1—C2—C3	−0.8 (3)	C14—C15—C19—C24	−178.82 (18)
S1—C1—C2—C3	−177.82 (18)	C16—C15—C19—C24	0.71 (18)
C1—C2—C3—C4	0.3 (4)	C14—C15—C19—C20	0.0 (3)
C2—C3—C4—C5	0.5 (4)	C16—C15—C19—C20	179.6 (2)
C3—C4—C5—C6	−0.7 (4)	C24—C19—C20—C21	0.7 (3)
C4—C5—C6—C1	0.2 (3)	C15—C19—C20—C21	−178.03 (19)
C2—C1—C6—C5	0.6 (3)	C19—C20—C21—C22	0.0 (3)
S1—C1—C6—C5	177.56 (16)	C20—C21—C22—C23	−0.4 (3)
C12—C7—C8—C9	−0.6 (3)	C21—C22—C23—C24	0.1 (3)
N1—C7—C8—C9	178.78 (18)	C22—C23—C24—C19	0.7 (3)
C7—C8—C9—C10	−1.0 (3)	C22—C23—C24—O3	179.87 (18)
C8—C9—C10—C11	1.4 (3)	C20—C19—C24—C23	−1.1 (3)
C9—C10—C11—C12	−0.1 (3)	C15—C19—C24—C23	177.98 (17)
C10—C11—C12—C7	−1.4 (3)	C20—C19—C24—O3	179.64 (15)
C10—C11—C12—C13	178.13 (18)	C15—C19—C24—O3	−1.28 (19)
C8—C7—C12—C11	1.7 (3)	C17—C18—N1—C7	179.74 (17)
N1—C7—C12—C11	−177.71 (15)	C13—C18—N1—C7	2.44 (18)
C8—C7—C12—C13	−177.86 (15)	C17—C18—N1—S1	−35.9 (2)
N1—C7—C12—C13	2.68 (19)	C13—C18—N1—S1	146.81 (13)
C11—C12—C13—C14	−0.1 (3)	C8—C7—N1—C18	177.44 (17)
C7—C12—C13—C14	179.43 (17)	C12—C7—N1—C18	−3.16 (18)
C11—C12—C13—C18	179.31 (18)	C8—C7—N1—S1	32.3 (2)
C7—C12—C13—C18	−1.15 (19)	C12—C7—N1—S1	−148.30 (13)
C18—C13—C14—C15	0.5 (2)	C17—C16—O3—C24	178.58 (16)
C12—C13—C14—C15	179.87 (16)	C15—C16—O3—C24	−0.83 (19)
C13—C14—C15—C16	−1.2 (2)	C23—C24—O3—C16	−177.93 (17)
C13—C14—C15—C19	178.28 (17)	C19—C24—O3—C16	1.32 (19)
C14—C15—C16—C17	0.3 (3)	C18—N1—S1—O1	169.12 (13)
C19—C15—C16—C17	−179.32 (17)	C7—N1—S1—O1	−51.20 (15)
C14—C15—C16—O3	179.70 (14)	C18—N1—S1—O2	40.22 (15)
C19—C15—C16—O3	0.08 (18)	C7—N1—S1—O2	179.89 (12)
O3—C16—C17—C18	−178.05 (15)	C18—N1—S1—C1	−75.31 (15)
C15—C16—C17—C18	1.3 (3)	C7—N1—S1—C1	64.37 (15)
C16—C17—C18—C13	−2.0 (3)	C6—C1—S1—O1	19.30 (19)
C16—C17—C18—N1	−178.95 (16)	C2—C1—S1—O1	−163.68 (15)
C14—C13—C18—C17	1.2 (3)	C6—C1—S1—O2	151.45 (16)
C12—C13—C18—C17	−178.32 (16)	C2—C1—S1—O2	−31.52 (18)
C14—C13—C18—N1	178.66 (14)	C6—C1—S1—N1	−94.69 (17)
C12—C13—C18—N1	−0.84 (18)	C2—C1—S1—N1	82.34 (17)
